# Fitness Facility Staff Can Be Trained to Deliver a Motivational Interviewing-Informed Diabetes Prevention Program

**DOI:** 10.3389/fpubh.2021.728612

**Published:** 2021-12-07

**Authors:** Tineke E. Dineen, Corliss Bean, Kaela D. Cranston, Megan M. MacPherson, Mary E. Jung

**Affiliations:** ^1^School of Health and Exercise Sciences, University of British Columbia, Kelowna, BC, Canada; ^2^Department of Recreational and Leisure Studies, Brock University, St. Catharines, ON, Canada

**Keywords:** training evaluation, implementation science (MeSH), health behavior (MeSH), prediabetic state, diet, exercise

## Abstract

**Background:** Training programs must be evaluated to understand whether the training was successful at enabling staff to implement a program with fidelity. This is especially important when the training has been translated to a new context. The aim of this community case study was to evaluate the effectiveness of the in-person Small Steps for Big Changes training for fitness facility staff using the 4-level Kirkpatrick training evaluation model.

**Methods:** Eight staff were trained to deliver the motivational interviewing-informed Small Steps for Big Changes program for individuals at risk of developing type 2 diabetes. Between August 2019 and March 2020, 32 clients enrolled in the program and were allocated to one of the eight staff. The Kirkpatrick 4-level training evaluation model was used to guide this research. Level one assessed staff satisfaction to the training on a 5-point scale. Level two assessed staff program knowledge and motivational interviewing knowledge/skills. Level three assessed staff behaviors by examining their use of motivational interviewing with each client. Level four assessed training outcomes using clients' perceived satisfaction with their staff and basic psychological needs support both on 7-point scales.

**Results:** Staff were satisfied with the training (*M* = 4.43; *SD* = 0.45; range = 3.86–4.71). All learning measures demonstrated high post-training scores that were retained at implementation follow-up. Staff used motivational interviewing skills in practice and delivered the program at a client-centered level (≥6; *M* = 6.34; *SD* = 0.83; range = 3.75–7.80). Overall, clients perceived staff supported their basic psychological needs (*M* = 6.55; *SD* = 0.64; range = 6.17–6.72) and reported high staff satisfaction scores (*M* = 6.88; *SD* = 0.33; range = 6–7).

**Conclusion:** The Small Steps for Big Changes training was successful and fitness facility staff delivered a motivational interviewing-informed program. While not all staff operated at a client-centered level, clients perceived their basic psychological needs to be supported. Findings support the training for future scale-up sites. Community fitness staff represent a feasible resource through which to run evidence-based counseling programs.

## Introduction

The prevalence of diabetes continues to rise, with 1 in 3 Canadians affected (1). Prediabetes acts as an early warning sign for type 2 diabetes (T2D), when individuals have blood glucose levels that are higher than normal, but not high enough to be diagnosed as T2D. This window of opportunity is important as individuals with prediabetes have up to 70% increased risk of developing T2D in the future (2). Large clinical trials have demonstrated that diet and exercise modifications are effective at reducing T2D risk by up to 58% in individuals with prediabetes (3), with risk-reducing effects lasting long after intervention end (4). Thus, a focus on reducing risks of developing T2D is critical to address this public health priority.

There have been multiple translations of T2D prevention programs into real-world settings with demonstrated effectiveness (5, 6). Many countries have developed large-scale translational studies [see (7) for an overview]. Despite the effectiveness of diabetes prevention programs in community settings (8), there remains a paucity of available programs in Canada. To reach and positively impact Canadians living at risk of T2D, effective programs need to be implemented in community settings and scaled-up across the country.

Several translational studies have been modeled on the United States Diabetes Prevention Program, adopting a group-based format. While group-based approaches demonstrate one method to reduce overall program costs (9), use of community members represents another. A recent systematic review indicated that overall, diabetes prevention programs are cost-effective in any setting, and the review contained no studies examining an individual format with community members (9). A systematic review assessing real-world impact of global diabetes prevention interventions identified 63 studies, of which only three adopted an in-person, individual approach with a health care provider (6). No studies adopted an in-person, individual approach led by a community member. More research on programs for reducing T2D risk using an individualized approach with community members, such as fitness facility staff, are needed.

Training is a core component of implementation with implications on program fidelity and subsequent effectiveness (10). Understanding the effectiveness of a training program, especially for fitness facility staff without experience in counseling and diabetes prevention, is integral to the development of scalable, cost-effective diabetes prevention programs. Assessing training is one of the five domains of the Behavioral Change Consortium's best practice guidelines for assessing fidelity in health behavior change trials (11). A training program must sufficiently teach the required knowledge and skills for attendees to be successful in implementing the program.

Motivational interviewing (MI) has been used in numerous health-related programs to help individuals change various health-related behaviors, including the management of diabetes and obesity (12, 13). When evaluated, MI training has been shown to be effective among health care practitioners (14). Evaluated training programs typically include a 1.5–3-day workshop with individual MI skill practice, role-play scenarios, and post-training support (14). To our knowledge no study to date has examined MI training for fitness facility staff, and few researchers have examined MI training for non-health care practitioners. Of the limited research, one study examined teaching community health agents to lead a T2D self-management program using a MI-informed approach in Brazilian primary care centers (15). The researchers examined MI fidelity and most community health agents performed at medium-to-high levels. However, MI fidelity was assessed using researcher-completed MI fidelity checklists, a less reliable method compared to audio or video recording sessions (16). The current study builds on previous work by examining the effectiveness of training fitness facility staff (hereafter referred to as staff) to deliver a MI-informed program using independently coded audio recordings to examine MI fidelity.

Recently, Phillips and Guarnaccia (17) recommended researchers who study MI could benefit from measuring self-determination theory (18) variables, such as basic psychological needs. Self-determination theory has been identified as a valuable theory to understand the mechanisms behind MI (19). There are three basic psychological needs: autonomy (i.e., client-centered approach, elicit client ideas), competence (i.e., confidence in the capacity to change), and relatedness (i.e., understand client perspective and non-authoritarian position) which overlap with core MI components (19). The learning climate questionnaire (20, 21) is a self-determination theory measure used to examine whether clients felt their basic psychological needs were met through program participation. To our knowledge, this is the first study to assess the learning climate questionnaire as an indicator of client perceptions of staff MI skills.

## Background and Rationale

The Small Steps for Big Changes (SSBC) program uses an individual approach to deliver behavior change techniques coupled with MI techniques to bolster client's self-regulatory skills in an autonomy supportive manner (22). The program's main behavior change techniques include goals and planning, feedback and monitoring, and repetition and substitution in relation to diet and exercise topics. The free program is housed in a community not-for-profit organization, the YMCA of Okanagan,^1^ and consists of six sessions over 3-weeks [for full program overview see (22)]. Prior to this current translation project, SSBC was running as a research project in a community site with research staff implementing the program. In the first 2 years, the program had 213 participants enrolled with a 95% completion rate (23). Clients demonstrated improved self-reported physical activity and dietary behaviors, improved distance on a 6-minute walk test, and reduced weight and waist circumference (23). To accelerate the benefit of this program reaching the 6 million Canadians at risk for developing T2D (24), translation to the community is necessary.

Translating the program to be run by a community organization presents an ideal opportunity to supplement gaps in primary healthcare and provide a cost-efficient program using YMCA staff. Hosting the program in a community organization has numerous benefits including increasing reach to those underserved by the healthcare system and enabling clients to have ongoing access to facilities (including their staff) post-program. The YMCA of Okanagan was actively looking for a program to adopt and inquired whether SSBC could be implemented within their facility (25). YMCA staff are well-suited to deliver such a program with their demonstrated interest in the health and fitness industry and expertise in physical activity (Dineen et al., unpublished). Through their established community presence, dedication to supporting healthy communities and availability of assisted membership post-program, the YMCA is an ideal partner to increase reach, accessibility, and long-term support. As part of a national organization, there are opportunities for scale-up across Canada. However, it is unknown if YMCA staff can effectively learn the program's counseling strategy (MI), diabetes prevention concepts and implement the program with fidelity. This current case study was needed to examine the effectiveness of training YMCA staff to implement a MI-informed diabetes prevention program.

As noted, SSBC centers on the delivery of diabetes prevention topics using MI skills and specific behavior change techniques (e.g., goal setting) to support clients' basic psychological needs and program success. The goal of the training was to teach staff core program components and skills necessary to be successful in facilitating the program to clients. The Kirkpatrick Evaluation Model (26) is a frequently used model to evaluate training programs. The model consists of four levels: (a) reaction, (b) learning, (c) behavior, and (d) results. The first level measures reactions to the training received. The second level measures the knowledge and skills learned. The third level measures whether the knowledge and skills acquired from the training are used in practice. The fourth level measures the effect from the use of the skills/information in practice. Despite the popularity of Kirkpatrick's evaluation model, all four levels are rarely assessed—with levels one and two being most reported [e.g., (27)]. Levels three and four are more challenging to measure as they require post-training follow-up to examine both the application of training skills in practice and the effect of those skills that were used. The current study delivers a comprehensive training evaluation using all four levels of the model. The purpose of this case was to evaluate the effectiveness of the in-person SSBC training using the Kirkpatrick 4-level training evaluation model. In line with this model, four research questions guided the case: (a) How satisfied are staff with the training?, (b) To what extent do staff learn the material taught in the training?, (c) To what extent do staff enact what they learned in practice?, (d) To what extent are clients satisfied with their staff and feel their basic psychological needs are supported?

### Case Description

A 1-year collaborative planning process with the YMCA of Okanagan was undertaken to support uptake and sustainability of the program (25). Two local YMCA sites were selected to implement the SSBC program and staff were invited to engage in the 1-year planning process. Following this planning process, interested staff volunteered to attend a 3-day (17-h) training workshop that covered MI principles, program content, and standard operating procedures. Two master trainers from the SSBC team (TD and KC) facilitated the training. The training workshop was previously developed and tested by the SSBC study team and refined prior to the current case (28, 29).

The workshop included didactic teaching of intervention content (e.g., effect of physical activity on diabetes prevention), a variety of role-play activities to practice use of skills (e.g., MI skills), demonstration of skills (e.g., exercise protocol), videos of session content delivered using MI skills, and how-to videos for common program procedures (e.g., how to review client's app-based diet and exercise tracking). All staff were given an implementation manual, including standard operating procedures, sample session scripts and checklists for the six sessions, and had ongoing access to the training videos through an online platform. All staff were shadowed by a research team member for their first client and given feedback after each session. Finally, ongoing monthly meetings with staff from each site were led by the first author to provide study updates, support, and review successes, challenges, and lessons learned.

Assessing training is an important fidelity assessment to gain confidence that staff are adequately trained to implement a program with high fidelity. This is a crucial step for effective program implementation, especially prior to scaling-up an intervention. Results will support refining a standardized training program for a community behavior change program for individuals at risk of T2D and support future scale-up of the training to other YMCAs.

## Methods

### Participants

#### Staff and Clients

All staff and clients provided written informed consent prior to participating. Staff who facilitated a minimum of three clients through the program were included in the present study (*n* = 8). Staff were, on average, 31.88 ± 9.05 years of age (range 24–51), predominantly self-identified as female (75%), and white (88%). There was a wide range in years of employment (50% had worked for 10+ years, 38% for 1–5 years, and 13% for <1 year) and education (75% had a university degree, 25% had obtained a college diploma) in staff. The eight staff facilitated clients (*n* = 32) through the program between August 2019 and March 2020. Clients were on average 59.56 ± 6.88 years of age (range 43–70), and predominantly self-identified as female (68%) and white (95%).

### Data Collection

Staff completed a pre-training, post-training, and implementation follow-up survey measuring various Kirkpatrick levels further described in the following sections. The pre- and post-training survey link were emailed to staff immediately before and after the 3-day workshop. The implementation follow-up survey link was emailed to staff after facilitating three participants through the program (*M* = 5 months post-training). Clients were emailed a pre-program survey link after their baseline appointment and a post-program survey link after completion of the program. All surveys were hosted on Qualtrics. The following sections describe how each level was measured and with what measure (see Table 1 for the measurement time-point table).

**Table 1:** Overview of measurement timepoints.

**Kirkpatrick level**	**Measurement**	**Timepoint**			
		**Staff pre-training**	**Staff post-training**	**Staff implementation follow-up**	**Client post-program**
1	Training satisfaction		✓		
2	Program knowledge test		✓	✓	
2	Motivational interviewing knowledge test		✓	✓	
2	Helpful response questionnaire	✓	✓	✓	
3	Motivational Interviewing Competency Assessment			✓^*^	
3	Program fidelity			✓*	
4	Learning climate questionnaire				✓
4	Satisfaction with staff				✓

* *Staff behaviors were assessed for every client they facilitated post-training*.

#### Level 1: Reaction

A post-training satisfaction survey was used to assess staff reaction to the training. A 12-item training satisfaction measure with demonstrated reliability was used [α = 0.88; (30)] whereby staff were asked to rate their confidence on a 5-point scale (totally disagree-totally agree; “In your opinion, the planned objectives were met”). Overall scale means were used in the analyses and demonstrated strong internal consistency (α = 0.89).

#### Level 2: Learning

To assess staff learning of program content and MI knowledge, two measures were assessed post-training and at implementation follow-up. First, staff knowledge of program content and standard operating procedures were assessed using a 39-item measure developed for this study. The measure was developed by the first author and pilot tested among the study team for readability, relevance, and suitability, resulting in minor modifications to questions to increase question comprehension (e.g., “How much sugar is recommended for women daily?”). Second, staff MI knowledge was assessed using a modified MI knowledge test (31). The original measure was modified from 23 to 17 items to reflect the MI content taught in the SSBC training. Responses to both knowledge measures were scored for each staff.

A third measure was used to assess staff knowledge of MI skills. The helpful response questionnaire (HRQ) is a brief measure to assess MI skills (32) and was administered to staff at pre-training, post-training and at implementation follow-up. Staff were asked to document how they would respond to six hypothetical patient statements. Staff were presented with the pre- and post-HRQ in person at the training workshop. The implementation follow-up HRQ was completed as part of the implementation follow-up online survey. An adapted scoring method was used for this study to reflect the level of MI taught and the skills in which staff were expected to be proficient. One point was given for a response with the presence of an MI communication skill (open-ended question, affirmation, and/or reflection) and zero points were given for a MI non-adherent response. A final score was calculated out of six. Two coders (TD and KC) scored all responses. Any disagreements were discussed by both coders.

#### Level 3: Behavior

To assess if staff used their program knowledge and skills in practice, an in-depth fidelity assessment was conducted and has been reported elsewhere [see (33)]. The study assessed whether staff implemented the program as intended by examining staff self-reported fidelity to session specific checklists and two independent coders cross-checked a sub-sample of audio-recorded sessions to ensure accuracy of staff self-report. In addition, goal setting fidelity (a key program component that relates to the goal setting and action planning behavior change techniques) was also examined.

In this current study, staff use of MI-specific skills and strategies were assessed using the MI Competency Assessment (MICA) (34). The MICA has been used in past research to assess use of MI during a session and has demonstrated reliability and validity (35). Two coders (KC and MM) listened to 20-minutes of one randomly selected program session for each client (*n* = 32). Coders rated the staff's use of five MI intentions (supporting autonomy and activation, guiding, expressing empathy, partnering, and evoking) and two MI strategies (strategically responding to change talk and strategically responding to sustain talk) on a 5-point scale from 1 (*inconsistent with MI*) to 5 (*proficient with MI*) for each audio recording. Items could be given a half-point. The five MI intentions aim to capture the overall spirit of MI. The coders also counted the frequency of two MI micro-skills (open-ended questions and reflections) within each coded session. A random 25% of sessions were double-coded. Based on the MICA double-coding standards (34), the coders discussed any disagreements and came to consensus.

#### Level 4: Training Outcomes

To assess training outcomes, two client measures were examined post-program that reflected staff program delivery. The first measure was the 15-item learning climate questionnaire (20, 21), which examined whether clients felt their basic psychological needs were met through program participation. The learning climate questionnaire has been used in various learning settings, such as within university and physical education contexts (20, 36). Three additional items were included to examine empathy, a key aspect of MI, including one item for each of the three elements of empathy: ability to understanding another's thoughts, feelings, and condition (e.g., “I felt the trainer was sensitive to my thoughts”). Clients were asked post-program to respond on a 7-point Likert scale (1 = strongly disagree; 7 = strongly agree). The learning climate questionnaire sub-scales (autonomy, competence, relatedness) demonstrated good internal consistency (α = 0.74–0.97) in addition to the empathy sub-scale (α = 0.91).

Finally, clients were asked to rate their overall satisfaction with their YMCA staff who facilitated them through the program. For this study, 1-item from a 10-item measure developed for this study on program quality (program and structures) was reported. The item measured overall staff satisfaction: “Please rate your satisfaction with your trainer?”. Staff satisfaction was chosen as a training outcome to represent whether clients felt their staff had delivered program components in a satisfactory manner. Clients were asked post-program to rate their confidence on a 7-point Likert scale from extremely dissatisfied to extremely satisfied. The entire 10-item program quality measure is available for reference in Supplementary File A.

### Data Analysis

A mean score was calculated for each measure and sub-scale. A paired-samples *t*-test was run to test for sustained effects on the program and MI knowledge measures, and the HRQ. As recommended, multiple agreement statistics were calculated for the HRQ including percent agreement, Cohen's kappa (K), and prevalence, and bias adjusted Kappa (PABAK) statistics (37, 38). For MICA coding, the five MI intentions were averaged and added to the average of the two MI strategies to have one overall score to represent each staff's use of the spirit of MI. Interclass Correlation Coefficients (ICC) were used to measure consistency in MICA coding across both coders (39). All MICA-subscales and the question to reflection ratio are averaged for each staff and presented in Supplementary File B. All measures demonstrated normality with the Shapiro-Wilks test and no outliers identified based on Tabachnick and Fidell's (40) criteria.

## Results

Overall, missing data for staff were low. One staff did not complete the post-training survey and one staff did not complete the implementation follow-up HRQ. Overall, post-program survey completion was moderate with 18 clients completing the learning climate questionnaire survey (56% response rate) and 17 clients completing the program quality survey (53% response rate).

### Level 1: Reaction

Table 2 presents the mean and standard deviation for staff satisfaction with the 3-day training. Overall, staff were satisfied with the training (*M* = 4.43, *SD* = 0.45). Staff agreed or totally agreed to 95% of all statements. Two statements had one staff responded neutrally to the items, “the topics were dealt with in enough detail and depth as the training allowed” and “the method of delivery was well-suited to the objectives and content.” Two staff disagreed to the item, “the length of the training was adequate for the objectives and content.”

**Table 2:** The mean and standard deviation for staff satisfaction with the 3-day training program.

**How would you rate the following items?**	**Mean (SD)**
In my opinion the planned objectives of the training were met	4.29 (0.49)
The topics were dealt with in enough detail and depth as the training allowed	4.29 (0.76)
The length of the training was adequate for the objectives and content	3.86 (1.35)
The method of delivery was well-suited to the objectives and content	4.29 (0.76)
The method of delivery used enabled us to take an active part in training	4.57 (0.54)
The training enabled me to interact/share experiences with my colleagues/others in the group	4.71 (0.49)
The training was realistic and practical	4.43 (0.54)
The resources provided were useful	4.43 (0.54)
The training context was well-suited to the training process	4.43 (0.54)
The training received is useful for my specific job	4.71 (0.49)
The training received is useful for my professional development	4.71 (0.49)
The training merits a good overall rating	4.43 (0.54)

*Scale ranged from 1 to 5*.

### Level 2: Learning

Similar trends occurred for all three learning measures; all demonstrated high post-training scores that were retained at implementation follow-up. A paired samples *t*-test [*t*_(6)_ = −0.13, *p* = 0.90] demonstrated staff's program knowledge test scores did not significantly differ between post-training (*M* = 18.74, *SD* = 2.20) and implementation follow-up (*M* = 18.84, *SD* =1.40). Similarly, a paired-samples *t*-test [*t*_(6)_ = −1.00, *p* = 0.36] demonstrated staff MI knowledge test scores did not significantly differ between post-training (*M* = 13.17, *SD* = 1.33) and implementation follow-up (*M* = 14.00, *SD* =1.41). Finally, a paired-samples *t*-test [*t*_(7)_ = −4.04, *p* = 0.01] demonstrated staff MI knowledge test scores significantly improved from pre- to post-training and there were no differences [*t*_(6)_ = −0.18, *p* = 0.86] between post-training and implementation follow-up (see Table 3 for individual staff HRQ scores). Overall, coders had high agreement on all consensus markers for HRQ coding (84.78% agreement, *K* = 0.64; PABAK = 0.70).

**Table 3:** Helpful response questionnaire (HRQ) and motivational interviewing competency assessment (MICA) scores per staff.

**Staff ID**	**Pre-training HRQ score (*n* = 8)**	**Post-training HRQ score (*n* = 8)**	**Implementation follow-up HRQ score (*n* = 7)**	**MICA Score *M* (SD) (*n* = 32)**
1	1	6	6	6.62 (0.42)
2	0	6	Missing	5.81 (0.56)
3	3	3	5	6.96 (0.55)
4	0	6	3	5.03 (1.11)
5	0	3	6	6.07 (0.15)
6	6	6	6	7.08 (0.39)
7	0	5	6	6.20 (0.74)
8	4	5	4	5.83 (0.88)
**Total** ***M*** **(SD)**	**1.75 (2.31)**	**5.00 (1.31)**	**5.14 (1.21)**	**6.34 (0.83)**

*HRQ score is out of 6; MICA score is out of 10 with a score of ≥ 6 indicating a client-centered level of care*.

### Level 3: Behavior

On average staff delivered the program at a client-centered level (*M* = 6.34, *SD* = 0.83; Range: 3.75–7.80). Based on staff availability, staff facilitated between 3 and 6 clients during the study timeframe. When assessed at the individual staff level, 5 staff operated above a client-centered level and 3 were below (see Table 3 for individual staff MICA scores). Overall, coders demonstrated excellent consistency with an ICC of 0.93.

### Level 4: Training Outcomes

Table 4 presents descriptive statistics for client responses to the learning climate questionnaire. Overall, clients perceived staff supported their basic psychological needs (*M* = 6.55, *SD* = 0.64). The mean for all the learning climate questionnaire sub-scales and the added empathy items obtained similar high results (A: 6.52, C: 6.47, R: 6.58, E: 6.67). Overall, clients reported high staff satisfaction scores (*M* = 6.88, *SD* = 0.33).

**Table 4:** The mean and standard deviation for client responses to the learning climate questionnaire.

**Sub-scale**	**Statement**	**Client**
A	I felt that the trainer provided me with choices and options	6.50 (0.86)
A	I felt understood by the trainer	6.50 (0.71)
A	I was able to be open with the trainer during the sessions.	6.61 (0.61)
A	The trainer showed confidence in my abilities to do well.	6.50 (0.79)
A	The trainer encouraged me to ask questions.	6.56 (0.71)
A	The trainer tried to understand how I'd see things before suggesting new ways to do things.	6.39 (0.98)
A	The trainer listened to how I would like to do things.	6.61 (0.61)
C	The trainer helped me to improve.	6.67 (0.59)
C	The trainer made me feel like I was good at exercise.	6.17 (1.54)
C	I felt that the trainer wanted me to do well.	6.72 (0.58)
C	The trainer made me feel like I was able to do the activities in the program.	6.33 (1.46)
R	The trainer supported me.	6.67 (0.59)
R	The trainer had respect for me.	6.67 (0.59)
R	The trainer was interested in me.	6.28 (1.49)
R	I felt that the trainer was friendly toward me.	6.71 (0.59)
E	I felt the trainer was sensitive to my thoughts	6.71 (0.59)
E	I felt the trainer was sensitive to my feelings	6.67 (0.59)
E	I felt the trainer was sensitive to my current life situation	6.65 (0.86)
	Autonomy sub-scale	6.52 (0.71)
	Competence sub-scale	6.47 (0.87)
	Relatedness sub-scale	6.58 (0.68)
	Empathy items	6.67 (0.63)

*Scale ranged from 1 to 7; A, autonomy; C, competence; R, relatedness; E, empathy*.

## Discussion

Training evaluations are an important component of assessing program fidelity and a vital implementation strategy during program translation and scale-up. Assessing training is a necessary factor to understand if a program was delivered as planned to have confidence in program outcomes (16). As a program is translated from being delivered by research staff to community members, researchers must examine whether the training is effective in the new context. Thus, the present study examined the SSBC training for YMCA fitness facility staff and had positive results. This evaluation was guided by the Kirkpatrick Evaluation Model (26), a model commonly used in training evaluations. Study strengths include evaluating all four levels of Kirkpatrick's model and using multiple perspectives. The training was effective for staff and demonstrated community members are a feasible resource to implement evidence-based counseling programs.

Past diabetes prevention programs have used MI [e.g., (41, 42)], including the original United States Diabetes Prevention Program (3). However, MI training and subsequent fidelity have not been explicitly evaluated in these programs. Rather, training effectiveness was determined using client outcomes as a proxy. In doing so, it is impossible to determine if staff were using MI skills in practice. One related study that targeted diet, exercise, or smoking behaviors among individuals at risk for diabetes and/or cardiovascular disease did include an MI evaluation (43). Practice nurses underwent 12 h of MI training, received a treatment manual and on the job coaching halfway through the intervention. Overall, nurses' skills in practice were low, despite their self-reported confidence. The researchers hypothesized the training was not long enough to improve counseling techniques and suggested the shift in practice (i.e., common for nurses to give advice, explain things to patients which is avoided when using MI) may require more time to implement. While the above examples took place in healthcare contexts, there remains a lack of research examining the use of MI in non-healthcare related fields. Prior research has demonstrated that the YMCA is a promising organization to disseminate a group-based diabetes prevention program achieving positive program outcomes (44). The current study extends this notion by demonstrating YMCA staff can learn MI during a 3-day (17-h) in-person training workshop and implement MI within their program sessions. When examining MI specific skills, staff HRQ increased from pre- to post-training and staff maintained their skills at the implementation follow-up timepoint, which is in line with previous MI training evaluations (14). Fitness facilities may be a promising community-based venue to deliver an MI-informed brief-counseling diabetes prevention program.

Although the fidelity of MI skills has not been examined in diabetes prevention programs, it has been examined in other fields. For pragmatic reasons, staff did not complete a pre-training audio recording of a counseling session. Therefore, comparisons of staff MICA scores from pre- to post-training were not done, which has been done in past training evaluations (4). Rather, the MICA tool was used to assess if staff reached the client-centered level of care, a less reported and valuable statistic. Overall, staff used MI skills in practice with an average MICA score of 6.3, indicating a client-centered level of care (≥6). When examining individual staff scores, some staff achieved higher MICA scores than others. Overall, five staff were operating above a client-centered level of care and three staff were operating close to, but below a client-centered level of care. These results are similar to a sample of community health agents from Brazil who underwent MI training. Overall, 13 community health agents were categorized as medium-or-high performance and 3 were categorized as low performance when MI fidelity was assessed using a researcher completed checklist of 10 MI techniques (15). It is important to recognize that some staff may learn and apply MI more quickly than others. Thus, more research is needed on how to move individuals operating at a level below client-centered care to a level at or above client-centered care, or how to effectively screen for competency prior to training.

Self-determination theory has been suggested as a potential conceptual framework for explaining how and why MI works (17, 19). While the learning climate questionnaire was developed for assessing the basic psychological needs (autonomy, competence, and relatedness), it has been argued that MI is a complementary approach that also aims to support closely related constructs (20, 31, 44), Therefore, in response to a recommendation in the literature (17), the learning climate questionnaire was used as a marker of MI use. To our knowledge, this is the first study to use the learning climate questionnaire to assess basic psychological needs in a MI study. Results demonstrated that clients felt supported by their staff on all three basic psychological need sub-scales and on the added empathy measure. Although some staff may not have reached a client-centered level of care on their MICA coded sessions, the staff were able to foster a positive leaning environment throughout the program that was perceived by clients as empathetic and supporting clients' autonomy, competence, and relatedness. Future research should consider similar analyses to assess MI use.

### Limitations

This research was affected by COVID-19 (YMCA and associated programming were forced to close due to the pandemic), is limited by a small sample size and results may lack generalizability. A pre-training knowledge test was not administered as all staff were naïve to working with individuals at risk for developing T2D and the program's standard operating procedures prior to the workshop. Similarly, there was no pre-training audio recorded session to capture staff pre-training MICA scores. These decisions were made for pragmatic reasons. In evaluation research, data collection methods (e.g., knowledge test) can act as part of the training to increase the likelihood that staff demonstrate a minimum level of knowledge gained (45). In doing so, after the evaluation is over, the data collection tools used remain a component of the training, increasing feasibility (i.e., reduce staff burden) and supporting future scale-up. All staff were naïve to MI prior to the training except one who completed a university level MI course. No staff had previous counseling experience. The HRQ gauged pre-training MI levels and indicated all staff had low levels of MI skills prior to the training, except the one staff with prior MI knowledge. In the current training, all staff had a research team member shadow all sessions with their first client and provide feedback post-session. As a conservative measure, these clients were included in the MICA scoring. In general MICA scores for the shadowed clients were consistent with future client scores. While not all staff reached a client-centered level, clients' perceived staff to support their basic psychological needs. The current study was pragmatic in nature and aimed to examine the training effectiveness. Therefore, all staff continued regardless of MI quality. On completion of the program, clients were sent a post-program survey through an email from the research team and had a moderate response rate (~50%). Future research should consider completing surveys during the final appointment or offer an incentive to increase response rates.

### Lessons Learned

Staff praised the shadowing experience for providing comfort during their first experience implementing the program and an opportunity to receive direct feedback on their program delivery. However, it was difficult to coordinate scheduling and is not feasible for scale-up. Similarly, finding time for all staff to attend a 3-day in-person training workshop was challenging due to conflicting staff work schedules. Translating the training into a self-paced digital training may better support scale-up and sustainability of the training for broad scale-out. Future research should examine novel ways to mimic the reverse shadow experience to adequately prepare staff, such as incorporating a mock session.

Conducting a training evaluation was invaluable to learn how the training was received and implemented by YMCA staff, and how clients were affected by their staff's execution of the program. The current study demonstrates that MI can be taught to fitness facility staff. Previous research has suggested MI was difficult to learn, with those struggling wishing to have more training (42). Indeed, the one item that two staff disagreed with related to training length. Interestingly, current results indicate that staff who complete training at a client-centered level of care, generally maintain that level post-training. Markers for staff competency or booster training sessions should be explored to better achieve a client-centered level of care for all staff prior to graduating the training.

## Conclusion

Implementing a program as it was intended to be implemented gives confidence that program outcomes are a result of the program structure and delivery (16). All staff attended a standardized training to learn how to implement the SSBC program effectively and reliably. Results demonstrated staff were satisfied with the training, developed the knowledge and skills to implement the program effectively, and applied the knowledge and skills in practice. In turn, clients felt their basic psychological needs were supported and were satisfied with their staff. While this study does not assess client outcomes, a successful training can positively affect clinical outcomes (46). Although client outcomes from the current study have been affected by the COVID-19 pandemic and are ongoing, evidence of program effectiveness when implemented by research staff is high (23). Current and prior results [i.e., (33)] indicate that staff implemented the program with fidelity which provides confidence that similar outcomes may result. Overall, this study demonstrated that the SSBC training was effective for fitness facility staff and is suitable to use for program scale-up. Future research should consider community members as a feasible resource to implement community-based counseling programs.

## Data Availability Statement

The raw data supporting the conclusions of this article will be made available by the authors, without undue reservation.

## Ethics Statement

The studies involving human participants were reviewed and approved by Conjoint Health Research Ethics Board, University of British Columbia: H16-02028. The patients/participants provided their written informed consent to participate in this study.

## Author Contributions

TD, CB, and MJ contributed to conception and design of the study. TD and KC completed the HRQ coding. KC and MM completed the MICA coding. TD organized the database, performed the statistical analysis, and wrote the first draft of the manuscript. All authors contributed to manuscript revision, read, and approved the submitted version.

## Funding

This research was funded by both a Social Sciences and Humanities Research Council Doctoral Scholarship (#767-2020-2130) and a Partnership Engage Grant (#892-2018-3065), the Canadian Institutes of Health Research (#333266), and Michael Smith Foundation for Health Research Reach Grant (#18120).

## Conflict of Interest

The authors declare that the research was conducted in the absence of any commercial or financial relationships that could be construed as a potential conflict of interest.

## Publisher's Note

All claims expressed in this article are solely those of the authors and do not necessarily represent those of their affiliated organizations, or those of the publisher, the editors and the reviewers. Any product that may be evaluated in this article, or claim that may be made by its manufacturer, is not guaranteed or endorsed by the publisher.

## Footnotes

^1^The YMCA waived their rights to anonymity.

## Abbreviations

T2D: Type 2 diabetes
SSBC: Small Steps for Big Changes
MI: Motivational interviewing
MICA: Motivational Interviewing Competency Assessment
HRQ: Helpful response questionnaire
ICC: Interclass correlation coefficients
PABAK: Prevalence and bias adjusted Kappa.
