# Supplementary material for: Fitness Facility Staff Can Be Trained to Deliver a Motivational Interviewing-Informed Diabetes Prevention Program
Source: Front Public Health. 2021 Dec 7;9:728612. doi: 10.3389/fpubh.2021.728612 (PMC8688685; doi:10.3389/fpubh.2021.728612)
Supplement: Supplementary file 1 [file Table_1.DOCX]

**Supplementary File A**

*Client satisfaction with program quality (program and structures)*

|  | Client |
| --- | --- |
| Your overall experience | 6.67 (0.49) |
| Your trainer | 6.88 (0.33) |
| The nutritional content | 6.17 (0.86) |
| The exercise content | 6.28 (0.96) |
| Having the program in a YMCA facility | 6.61 (0.61) |
| The program workbook | 6.00 (1.00) |
| The Health Watch 360 app | 6.17 (0.99) |
| Receiving a target heart rate zone | 6.28 (1.18) |
| The length of sessions | 6.44 (0.98) |
| The program length | 5.83 (1.62) |
| *Note.* Scale ranged from 1-7 | |
