# Supplementary material for: Fitness Facility Staff Can Be Trained to Deliver a Motivational Interviewing-Informed Diabetes Prevention Program
Source: Front Public Health. 2021 Dec 7;9:728612. doi: 10.3389/fpubh.2021.728612 (PMC8688685; doi:10.3389/fpubh.2021.728612)
Supplement: Supplementary file 2 [file Table_2.DOCX]

**Supplementary File B**

*Average Motivational Interviewing Competency Assessment (MICA) scores by sub-scale per staff*

| Staff ID | Ratio of R:Q | Sustain talk | Change talk | Autonomy | Guiding | Empathy | Partnership | Evoking | **Total MICA Score** |
| --- | --- | --- | --- | --- | --- | --- | --- | --- | --- |
| 1 (*n* = 6) | 0.50 (0.22) | 3.08 (0.20) | 3.21 (0.33) | 3.46 (0.33) | 3.63 (0.38) | 3.38 (0.21) | 3.63 (0.31) | 3.29 (0.25) | **6.62 (0.42)** |
| 2 (*n* = 3) | 0.37 (0.06) | 3.00 (0.00) | 2.75 (0.43) | 3.08 (0.38) | 2.50 (0.87) | 3.00 (0.00) | 3.08 (0.38) | 3.00 (0.43) | **5.81 (0.56)** |
| 3 (*n* = 6) | 1.32 (1.24) | 3.17 (0.41) | 3.38 (0.44) | 3.54 (0.33) | 3.17 (0.75) | 3.46 (0.33) | 3.63 (0.38) | 3.42 (0.41) | **6.96 (0.55)** |
| 4 (*n* = 3) | 0.27 (0.17) | 2.50 (0.50) | 2.67 (0.58) | 2.50 (0.50) | 2.42 (0.63) | 2.58 (0.72) | 2.58 (0.72) | 2.17 (0.58) | **5.03 (1.11)** |
| 5 (*n* = 3) | 0.64 (0.15) | 3.00 (0.00) | 3.00 (0.00) | 3.17 (0.29) | 3.08 (0.14) | 3.00 (0.00) | 3.08 (0.38) | 3.17 (0.29) | **6.07 (0.15)** |
| 6 (*n* = 4) | 0.94 (0.65) | 3.13 (0.25) | 3.44 (0.43) | 3.63 (0.25) | 4.00 (0.00) | 3.75 (0.29) | 3.81 (0.24) | 3.81 (0.24) | **7.08 (0.39)** |
| 7 (*n* = 4) | 0.34 (0.41) | 3.00 (0.00) | 3.13 (0.25) | 3.19 (0.63) | 3.31 (0.63) | 3.06 (0.66) | 3.06 (0.66) | 3.06 (0.66) | **6.20 (0.74)** |
| 8 (*n* = 3) | 0.85 (0.67) | 3.00 (0.00) | 2.83 (0.58) | 3.17 (0.76) | 2.83 (0.29) | 2.92 (0.52) | 2.83 (0.58) | 2.83 (1.04) | **5.83 (0.88)** |
| **Total**  **M (SD)**  (*n* = 32) | **0.70 (0.70)** | **3.02 (0.30)** | **3.11 (0.44)** | **3.28 (0.50)** | **3.20 (0.69)** | **3.21 (0.49)** | **3.30 (0.57)** | **3.16 (0.61)** | **6.34 (0.83)** |
| *Note.* R:Q = reflection to question ratio; All sub-scales are out of 5 with a score of ≥ 6 indicating a client-centered level of care; Total MICA score is out of 10 with a score of ≥ 6 indicating a client-centered level of care. | | | | | | | | | |
